# A GTP-synthesizing ribozyme selected by metabolic coupling to an RNA polymerase ribozyme

**DOI:** 10.1126/sciadv.abj7487

**Published:** 2021-10-06

**Authors:** Arvin Akoopie, Joshua T. Arriola, Douglas Magde, Ulrich F. Müller

**Affiliations:** Department of Chemistry and Biochemistry, University of California San Diego, La Jolla, CA 92093, USA.

## Abstract

Synthesis of RNA in early life forms required chemically activated nucleotides, perhaps in the same form of nucleoside 5′-triphosphates (NTPs) as in the contemporary biosphere. We show the development of a catalytic RNA (ribozyme) that generates the nucleoside triphosphate guanosine 5′-triphosphate (GTP) from the nucleoside guanosine and the prebiotically plausible cyclic trimetaphosphate. Ribozymes were selected from 1.6 × 10^14^ different randomized sequences by metabolically coupling 6-thio GTP synthesis to primer extension by an RNA polymerase ribozyme within 10^16^ emulsion droplets. Several functional RNAs were identified, one of which was characterized in more detail. Under optimized reaction conditions, this ribozyme produced GTP at a rate 18,000-fold higher than the uncatalyzed rate, with a turnover of 1.7-fold, and supported the incorporation of GTP into RNA oligomers in tandem with an RNA polymerase ribozyme. These results are discussed in the context of early life forms.

## Teaser

An RNA that catalyzes the formation of GTP was developed with an emulsion-based in vitro selection system.

## INTRODUCTION

Early life forms likely used catalytic RNAs in central roles before the evolution of encoded protein synthesis (*1*), as judged by the existence of the ribosome (*2*) and ribonuclease (RNase) P (*3*) in every known biological organism. To investigate what functions ribozymes could have fulfilled in early stages of life, in vitro selection experiments (*4*, *5*) were developed to generate ribozymes with functions that seem important for such molecular systems (*6*). One such ribozyme catalyzes template-dependent RNA polymerization using nucleoside 5′-triphosphates (NTPs), with recent ribozyme variants that are capable of synthesizing functional ribozymes (*7*–*9*).

NTPs were likely important in early life because they are used in every known biological organism as the central energy currency and as activated monomers for RNA polymerization, among many other roles (*10*). As activating agents, polyphosphates can be formed by several different prebiotically available routes (*11*–*15*). The prebiotic existence of phosphite—an intermediate in several of these routes—has been confirmed by its direct identification in 3.5-billion-year-old marine sediments (*16*). The most reactive polyphosphate is cyclic trimetaphosphate (cTmp) (*17*), which can react with nucleoside 5′-hydroxyl groups to form NTPs (*18*–*20*). However, the rate of the uncatalyzed reaction is low, with an observed rate of 4 × 10^−5^ M^−1^ hour^−1^ for adenosine 5′-triphosphate (ATP) at 1 mM adenosine, 10 mM cTmp (pH 7), 150 mM MgCl_2_, and 20°C; the rate for GTP is about threefold lower (*21*). While this rate increases with higher pH and temperature, those conditions also decrease the lifetime of RNA polymers. Therefore, there would have been an evolutionary benefit for ribozymes to catalyze the 5′-triphosphorylation of nucleosides at neutral pH and moderate temperature. In vitro selection experiments have shown that some ribozymes are able to catalyze the chemistry of this reaction, but those ribozymes catalyze the 5′-triphosphorylation of RNA oligomers (*22*) and do not generate free NTPs.

**Fig. 1.**
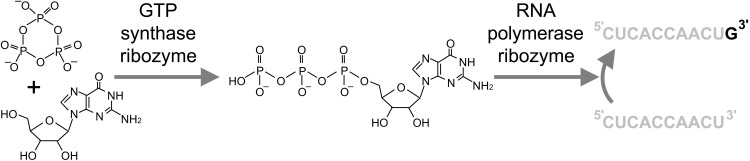
Metabolic coupling of two ribozymes by GTP. The reaction between cTmp (top left) and guanosine (bottom left) is catalyzed by a ribozyme developed in this study (GTP synthase, GTR1). The resulting GTP is used by a polymerase ribozyme to extend the 3′-terminus of an RNA primer (gray).

Ribozymes that generate free NTPs are central for origin-of-life model systems based on catalytic RNAs. The reason is that any metabolism requires metabolites that can freely be exchanged between multiple catalysts within a compartment, such as a cell or a protocell. However, the development of such coupled catalysts by in vitro selection is difficult because freely diffusing metabolites—in this case, NTPs—can escape the ribozymes that generated them and therefore not tag the active RNA sequence for selection. To overcome this hurdle, we established a coupled in vitro selection system in emulsion, where active sequences were tagged within the emulsion droplets by a second ribozyme. In this coupled system, one ribozyme generated guanosine 5′-triphosphate (GTP) from guanosine and cTmp, and a second ribozyme used the GTP to extend an RNA polymer (Fig. 1). Thereby, this study represents an important advance in establishing a metabolism for an RNA-based model system of early life.

## RESULTS

### In vitro selection

**Fig. 2.**
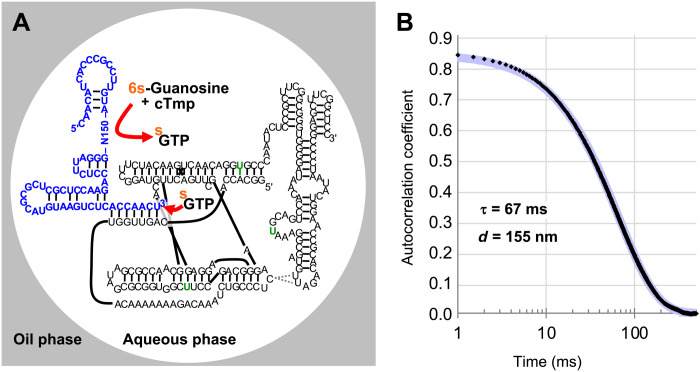
In emulsio selection setup. (**A**) Secondary structure representation of the in emulsio selection setup. The emulsion consisted of an oil phase matrix (gray) and aqueous droplets (white). Two RNA strands were base-paired to each other within the droplet. The pool RNA (blue) contained 150 nucleotides of a randomized region (N150) and a 3′-terminus that base-paired to a polymerase ribozyme (black). Desired pool molecules catalyzed the reaction of 6sGsn with cTmp to form 6sGTP (upper red arrow). The polymerase ribozyme then used 6sGTP to extend the pool 3′-terminus, thereby thio-tagging the 3′-terminus of the pool RNA (lower red arrow). Three mutations in the polymerase ribozyme increased the rate of 6sGTP ligation (green letters). The secondary structure of the pool construct terminus was predicted by mfold (*32*). The secondary structure of the polymerase ribozyme is based on previous studies (*33*, *34*). (**B**) Autocorrelation function from dynamic light scattering (DLS) analysis of the emulsion used in the selection. The autocorrelation values are plotted as a function of time, with time displayed logarithmically. The data points are shown as small black diamonds. A single-exponential fit (blue) had a time constant of 67 ms, correlating to an aqueous droplet diameter of 155 nm.

An in vitro selection procedure was designed to obtain ribozymes that catalyze the triphosphorylation of 6-thio guanosine (6sGsn) with cTmp to yield 6′-thio guanosine triphosphate (6sGTP) (Fig. 2A). The RNA library was prepared with a 5′-hydroxyl group, a 5′-terminal constant region, 150 nucleotides of randomized region, a 3′-terminal constant region, and a recognition site for a DNAzyme. To track these pool molecules, a fraction of them were 5′-[^32^P]–radiolabeled. Because T7 RNA polymerase generates heterogeneous 3′-termini (*23*–*25*) and a precise 3′-terminus with 2′,3′-diols was required for the selection procedure, transcribed RNA pool molecules were processed with a catalytic DNA that generates a 2′,3′-hydroxyl terminus with a defined length (*26*). The resulting gel-purified, weakly radiolabeled pool molecules were used for the selection step. To mediate tagging of RNA pool molecules that catalyzed the reaction of 6sGsn with cTmp to 6sGTP, the pool molecules were heat-renatured with an excess of a polymerase ribozyme variant that had been selected to efficiently use 6sGTP for tagging of an RNA 3′-terminus (*27*).

To compartmentalize individual pool molecules, the aqueous solution was emulsified in an oil phase containing mineral oil and the emulsifier ABIL EM 90 (*28*) at final concentrations of 0.5 μM RNA pool, 3 μM polymerase ribozyme, 50 mM tris-HCl (pH 8.3), 150 mM MgCl_2_, 200 mM KCl, 50 mM cTmp, and 1 mM 6sGsn. The emulsion droplet diameter was in the range of 150 nm (Fig. 2B), which resulted in an average of 0.5 RNA pool molecules and 3 polymerase ribozyme molecules per droplet. This droplet diameter also ensured that the generation of a single molecule of 6sGTP would result in 6sGTP concentration of about 1 μM in that particular droplet. Because the polymerase ribozyme used in this study displayed a 6sGTP ligation rate of 0.012 min^−1^ at 1 μM 6sGTP concentration (*27*), the production of a single 6sGTP molecule was expected to lead to the 3′-tagging of 50% of the co-compartmentalized pool molecules in less than 1 hour. The emulsion was incubated for 6 hours, and the RNA molecules were extracted.

To isolate those pool RNA molecules that were tagged with 6sGTP at their 3′-terminus, they were separated from untagged RNAs by three-layered aminophenyl mercury (APM)–polyacrylamide gel electrophoresis (PAGE) (*29*, *30*). The thio modification of 6sGTP efficiently immobilized the active pool molecules at the mercury interface during APM-PAGE, as observed in a previous in vitro selection experiment using 6sGsn (*31*). After the RNA pool molecules were isolated from the APM interface using dithiothreitol (DTT), they were reverse-transcribed, polymerase chain reaction (PCR)–amplified, and appended with overhanging PCR primers to regenerate the pool, now enriched for active sequences.

### Progress of the in emulsio selection

During the selection, several parameters were adjusted (fig. S1). In the first round of selection, the highest concentration of pool RNA molecules was used (500 nM) to be able to cover a large sequence space (1.6 × 10^14^ independent sequences). An average of 30 copies were used for each pool molecule sequence, corresponding to a total of 5 × 10^15^ molecules. Because the emulsion in the first round of selection contained 10^16^ droplets, this means that every second droplet contained one pool molecule, on average. In subsequent rounds of selection, the concentration of pool RNA was successively decreased to 50 nM to reduce the likelihood of selecting for interacting pool molecules. The concentration of the polymerase ribozyme was kept constant so that, in all rounds, statistically about 95% of the emulsion droplets contained at least one polymerase ribozyme molecule. The first selection round used 1 mM 6sGsn, about half of its solubility limit, and 50 mM cTmp, to capture even weakly active pool molecules. To enrich for active pool sequences with increased affinity to 6sGsn and cTmp, the concentrations were successively reduced to 50 μM 6sGsn and 0.5 mM cTmp.

**Fig. 3.**
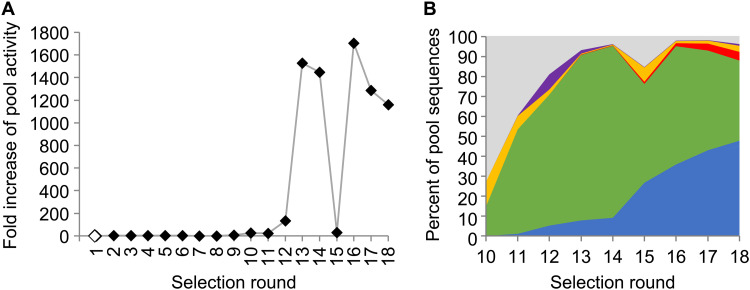
Progress of the in emulsio selection. (**A**) Progress of the selection, as judged by the pool activity as a function of the selection round. Pool activity was measured as the fraction of the radiolabeled pool that was retained at the APM-PAGE interface. To account for the decreased concentrations of cTmp and 6sGsn over the selection rounds, the retained pool fraction was multiplied by both decreases in concentration. (**B**) Abundance of sequence clusters as judged by HTS analysis of the selected pools. Sequence clusters are indicated by color, with clusters 1 (blue), 2 (green), 3 (red), 4 (yellow), and 5 (purple) and unassigned sequences (gray). Selection round 15 used mutagenic PCR, correlated with a decrease in activity that recovered in round 16.

The average 6sGsn triphosphorylation activity of the RNA pool during the selection was monitored by 5′-[^32^P]–radiolabeling a fraction of the pool and phosphorimaging of the APM gels. By measuring the fraction of pool molecules captured at the APM interface, and normalizing for the concentrations of 6sGsn and cTmp, an average pool ligation rate was calculated and plotted as a function of the selection rounds (Fig. 3A). After 12 cycles of selection, the pool showed a marked increase in activity, to about 1500-fold above the background rate of the reaction. Because activity plateaued in selection round 14, mutagenic PCR was used in round 15 to explore sequence variants of the initially selected sequences. The corresponding reduction in pool activity in round 15 was followed by continued, high activity in selection rounds 16 to 18.

To identify active sequences, the pools of all selection rounds were subjected to high-throughput sequencing (HTS) analysis (Fig. 3B). About 400,000 sequences were analyzed for each selection round, and sequences that showed more than 75% sequence identity were clustered. The resulting data show that the pool was dominated by five clusters during the last rounds of selection. The most active sequence variants within each of the active clusters were identified by the highest enrichment of their mutations over the last five rounds of the selection (fig. S2) (*35*). On the basis of these data, four of the most promising sequences were chosen from clusters 1 to 3, and the two most promising sequences from clusters 4 and 5. One additional sequence was generated each for clusters 2 and 4, which combined multiple enriched mutations.

### Biochemical analysis of selected sequences

**Fig. 4.**
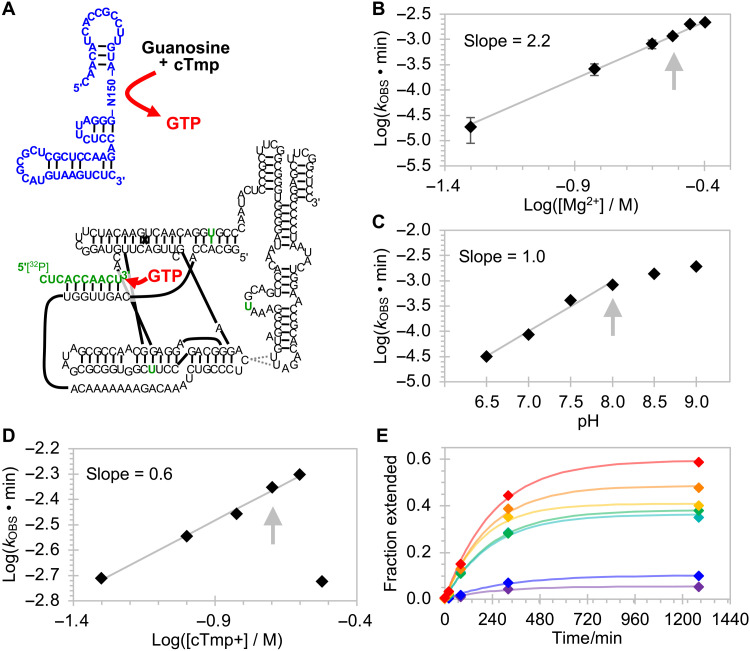
Optimization of reaction conditions for GTR1. (**A**) Schematic for the trans-activity assay. The isolated pool sequence (blue) was tested for catalyzing the formation of GTP (red), physically separated from the polymerase ribozyme (black) with mutations for enhanced GTP processing (green). A 5′-radiolabeled RNA 10-mer (green) was extended by the polymerase ribozyme from 10 to 11 nucleotides, and the 10-mer and 11-mer were separated by PAGE and quantified by phosphorimaging. The fraction of elongated RNA was determined for reaction time courses, and the reaction rates were determined by single-exponential curve fitting. These rates are plotted as a function of the reaction conditions. Starting from an initial condition of 125 mM Mg^2+^, 100 mM KCl, 50 mM tris-HCl (pH 8.3), 22°C, 25 mM cTmp, 1 mM Gsn, and 1 μM ribozyme, the conditions were successively optimized to (**B**) 300 mM Mg^2+^ (error bars are SDs from three experiments), 1 M K^+^, (**C**) pH 8.0, 22°C, (**D**) 200 mM cTmp, (**E**) 2 mM guanosine, and 9 μM GTR1. Graphs not shown in this figure appear in fig. S6. Gray arrows indicate the chosen conditions. The gray lines show linear least-squares fits to data points, with slopes given in the inserts. In (E), the guanosine concentrations are 0.1 mM (purple), 0.2 mM (blue), 0.5 mM (teal), 0.75 mM (green), 1 mM (yellow), 1.5 mM (orange), and 2 mM (red), and the colored lines are least-squares fits of single-exponential functions to the data of the corresponding color.

To analyze the chosen sequences for their generation of freely diffusing NTPs, an assay was developed in which the produced NTP was used to extend the 3′-terminus of a 5′-radiolabeled RNA 10-mer (Fig. 4A). At the same time, the pool molecules were truncated at their 3′-termini so that they would not base pair to the polymerase ribozyme. In this format, the NTP molecules generated by triphosphorylation ribozymes were required to diffuse from one ribozyme to the other. The reaction products were separated by PAGE, and the fraction of extended radiolabeled RNAs was measured.

When the ribozymes were challenged to triphosphorylate 6sGsn, the strongest signal for primer extension was seen for ribozymes from cluster 5 (fig. S3, A and B). In contrast, the formation of GTP from guanosine and trimetaphosphate showed the most efficient primer extension with ribozymes from cluster 1 (fig. S3, C and D). Sequence 59 of cluster 1 showed the strongest average signal. When this sequence was truncated at the 5′-terminus or the 3′-terminus, the signal dropped markedly, whereas an internal region of the ribozyme (nucleotides 47 to 76) could be removed without a loss in signal (fig. S4). The resulting ribozyme was termed GTR1 (guanosine triphosphorylation ribozyme 1), had a length of 175 nucleotides, and was chosen for further analysis.

### Characterization of the winning ribozyme GTR1

For a more detailed biochemical analysis of GTR1, the assay was modified to quench the GTR1 ribozyme after incubation with guanosine and cTmp before the detection of GTP (fig. S5). This allowed recording reaction kinetics for the GTP synthesis reaction and optimizing the reaction conditions (Fig. 4 and fig. S6). The dependence of the observed rate on the Mg^2+^ concentration showed a log-linear relation with a slope of 2.2, suggesting that 2 or 3 magnesium ions are limiting for the reaction kinetics (*36*). The dependence on pH showed a slope of 1.0, consistent with a single deprotonation step as the rate-limiting step in the reaction. This is likely the deprotonation of the nucleoside 5′-hydroxyl group, analogous to our earlier studies on self-triphosphorylation ribozymes (*22*). The dependence on cTmp concentration at 300 mM Mg^2+^ was consistent with Mg^2+^ forming a 1:1 complex with cTmp and leaving additional free Mg^2+^, as observed for self-triphosphorylating ribozymes (*22*). An increase in the guanosine concentration did not lead to a substantial increase in the triphosphorylation rate but to an increase in the amplitude. The chosen conditions were 300 mM MgCl_2_, 1 M KCl, 50 mM tris-HCl (pH 8.0), 22°C, 200 mM cTmp, 2 mM guanosine, and 9 μM GTR1.

**Fig. 5.**
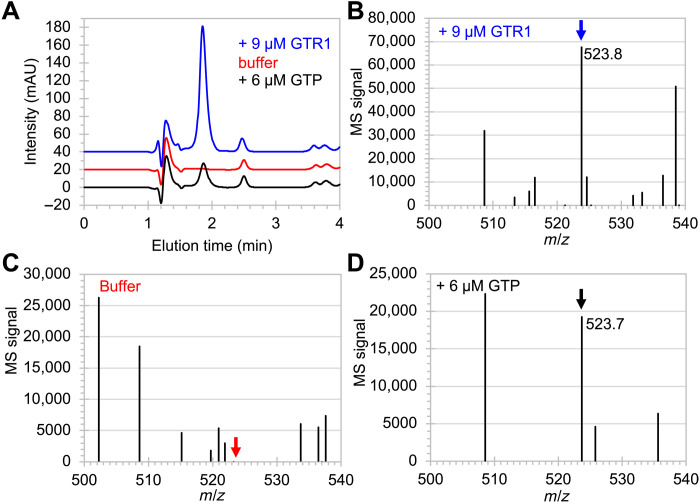
LC-MS analysis of reaction products between guanosine and cTmp. (**A**) Trace at 260 nm during reversed-phase HPLC of reaction products in reaction buffer containing guanosine and cTmp. In the presence of 9 μM GTR1 (blue trace), a strong peak at the elution time of 1.9 min resulted, which was absent without added ribozyme (red trace) and appeared when 6 μM GTP was added to the reaction buffer (black trace). mAU, milli-absorption units at 260 nm. Positive ion mode ESI-MS at the elution time of 1.9 min resulted in (**B**) a signal at *m*/*z* of 523.8 when GTR1 was present (blue arrow), (**C**) no such signal when the ribozyme was absent (red arrow), and (**D**) a signal at 523.7 when 6 μM GTP was added to the reaction buffer (black arrow).

To test whether GTP was produced by GTR1, the reaction products were analyzed by liquid chromatography (LC), followed by mass spectrometry (MS). During reversed-phased high-performance liquid chromatography (HPLC; Fig. 5A), a peak at elution time at 1.9 min was visible when reaction buffer containing guanosine and cTmp was incubated overnight with 9 μM GTR1 but not when the ribozyme was absent. The peak appeared again when 6 μM GTP was added to the reaction buffer. When this peak was analyzed by electrospray ionization (ESI)–MS in positive ion mode, the sample with 9 μM GTR1 (Fig. 5B) showed a strong peak with mass/charge ratio (*m*/*z*) of 523.8, as expected for GTP (calculated 524.2). This signal was absent for the sample without GTR1 (Fig. 5C) and appeared again in the sample containing 6 μM GTP (Fig. 5D). The fragmentation patterns of the peak around *m*/*z* of 524 gave *m*/*z* values of 152.2 and 363.8 for the sample containing GTP, and *m*/*z* values of 152.2 and 363.9 for the sample incubated with GTR1 (fig. S7). The sample containing only reaction buffer did not show these signals. These results confirmed that GTR1 catalyzed the formation of GTP.

**Fig. 6.**
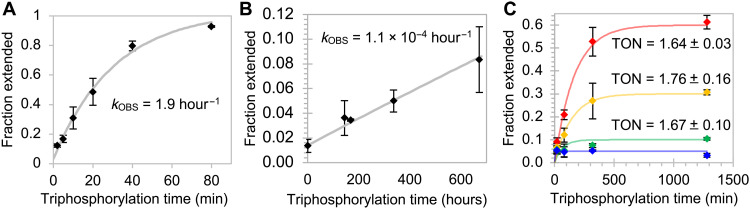
Rate enhancement and turnover of ribozyme-catalyzed GTP synthesis. The rates of the reaction catalyzed by 9 μM GTR1 (**A**) and the uncatalyzed reaction (**B**) were obtained by single-exponential fits (gray) to the kinetic data (diamonds). Note the difference in the scales of the axes. Error bars are SDs from triplicate experiments. (**C**) Determination of turnover number with a fixed concentration of radiolabeled primer with increasing concentrations of GTR1. The GTR1 concentration was 6 μM (red), 3 μM (yellow), 1 μM (green), or 0 μM (blue). The ratio of extended primer over GTR1 after ~1280 hours equaled the turnover number (TON), which is given as inserts.

To determine the catalytic rate enhancement by the GTR1 ribozyme, the formation of GTP from guanosine and cTmp was measured under optimal conditions as determined above (Fig. 6A). The rate for the catalyzed reaction with 9 μM GTR1 was 1.9 hour^−1^, compared to a rate of 1.1 × 10^−4^ hour^−1^ for the uncatalyzed reaction under the same conditions but without GTR1 (Fig. 6B). This corresponded to an 18,000-fold rate enhancement. The turnover number of the ribozyme was determined by reacting varying concentrations of GTR1 with a fixed concentration of substrate that was elongated with the produced GTP (Fig. 6C). The end points of the reaction kinetics showed that each GTR1 ribozyme molecule produced, on average, only 1.7 GTP molecules under these conditions. This number is a lower estimate because not every generated GTP molecule is detected by extension of the primer.

**Fig. 7.**
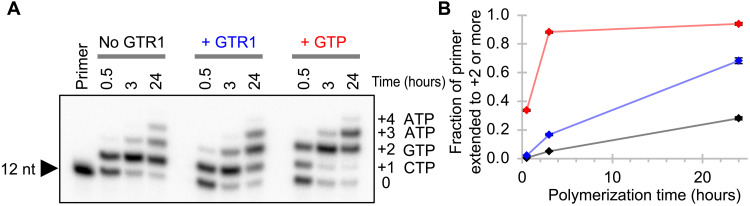
Ribozyme-mediated RNA polymerization coupled to ribozyme-catalyzed GTP synthesis. (**A**) Autoradiogram of polymerization products separated by denaturing 20% PAGE. The first reaction lacked GTP and GTR1 (No GTR1, black), the second reaction lacked GTP but contained the products from a GTP synthesis reaction with GTR1 (+ GTR1, blue), and the third reaction contained GTP (+ GTP, blue). For each of the three different reactions, samples from three polymerization time points were analyzed (0.5, 3, and 24 hours). The identity of the templated NTP is shown on the right for nucleotide (nt) additions +1 to +4. (**B**) Quantification of extension products. The fraction of primers extended by at least two nucleotides is plotted as a function of reaction time for three different conditions described in (A). Error bars are SDs from three experiments and mostly smaller than the symbols. The values at 24-hour polymerization time are 28.1 ± 1.1% (no GTR), 68.3 ± 2.0% (+ GTR), and 94.0 ± 0.9% (+ GTP).

We tested whether GTP formed by the GTR1 ribozyme could be incorporated into an RNA polymer using an RNA polymerase ribozyme (Fig. 7A). The template dictated the extension by cytidine 5′-triphosphate (CTP), GTP, and ATP, but only CTP and ATP were added as NTPs. In contrast, GTP (position +2) was provided from a GTR1-catalyzed guanosine triphosphorylation reaction. Quantification of the reaction product pattern (Fig. 7B) and comparison with the same reaction lacking GTR1 confirmed that most of the nucleotides incorporated at position +2 originated from catalysis by GTR1. The background rate without GTR1 likely resulted from misincorporation by the polymerase ribozyme (*7*). These results confirmed that GTR1-generated GTP can be incorporated during ribozyme-mediated RNA polymerization.

**Fig. 8.**
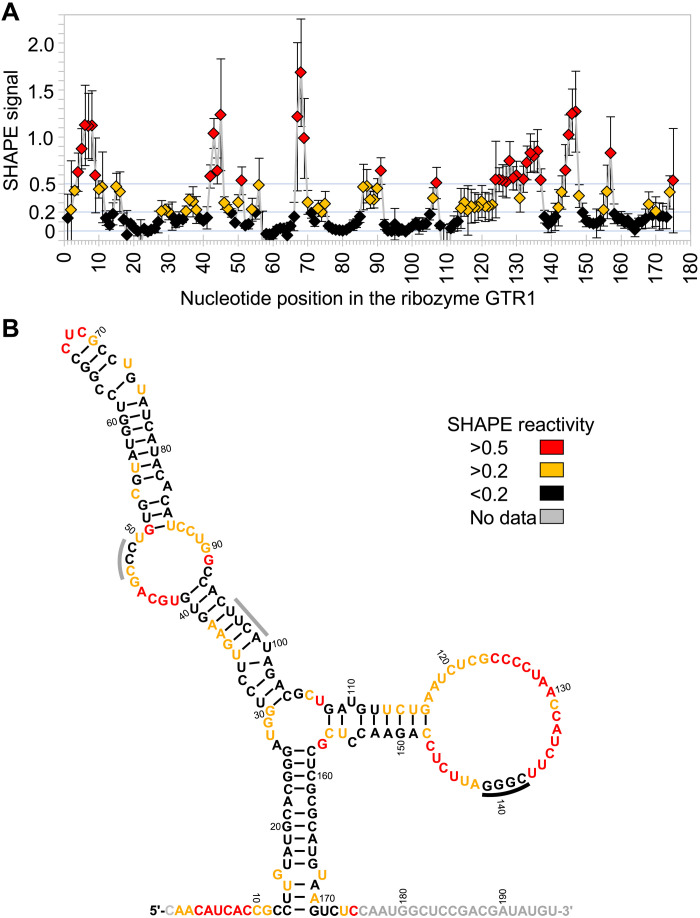
Secondary structure analysis of the ribozyme GTR1. (**A**) SHAPE reactivity profile of the GTR1 ribozyme under optimal reaction conditions. The SHAPE reactivity was determined by reverse transcription of two radiolabeled primers with the SHAPE-reacted RNA. Cutoffs of 0.2 and 0.5 (light blue lines) were chosen to distinguish positions with low reactivity (black), medium reactivity (orange), and high reactivity (red). Error bars are SDs from three independent probing experiments. (**B**) Secondary structure resulting from calculation with RNAfold in the ViennaRNA package 2.0 (*38*), using the constraints of the SHAPE reactivity. Color coding is as in (A). The black bar at positions 138 to 141 denote a SHAPE-protected region that may pair with positions 47 to 49 or 86 to 89 (gray bars). Positions labeled in gray did not have SHAPE signals because a signal corresponding to position 1 was too close to full-length extension and because positions 176 to 195 were covered by the reverse transcription primer.

The secondary structure of GTR1 was analyzed using SHAPE (selective 2′ hydroxyl acylation analyzed by primer extension) probing with 1-methyl-7-nitroisatoic anhydride (1M7) (*37*) at the optimal ribozyme reaction conditions, with tris-HCl buffer replaced by Hepes-NaOH (Fig. 8). The SHAPE reaction products were analyzed by reverse transcription with a 5′-[^32^P]–radiolabeled primers and separation by denaturing PAGE. To allow probing the complete sequence of the ribozyme, the ribozyme was extended by 20 nucleotides that did not interfere with the ribozyme’s function (gray 3′-tail in Fig. 8B). The protection pattern suggested a secondary structure with a central, tri-helical junction. Tertiary contacts appear to form between the protected nucleotides 138 to 141 and either nucleotide positions 47 to 49 or 96 to 99. Future structural analysis and sequence optimization will reveal how this ribozyme is able to catalyze the reaction between guanosine and cTmp to GTP.

## DISCUSSION

This study demonstrated a de novo in emulsio selection of ribozymes from random sequence. In vitro selection in emulsion has been used previously for selecting trans-active RNAs. However, these previous selections used the emulsion system to optimize a previously selected ribozyme. One study (*39*) selected variants of the previously developed class I ligase ribozyme (*40*), one study (*41*) selected variants of a previously selected Diels-Alderase ribozyme (*42*), and two studies optimized the polymerase ribozyme (*7*) for higher polymerization efficiency (*43*, *44*). These studies relied on in vitro selection experiments in bulk to generate ribozymes and later improved their performance by subsequent evolution rounds in emulsion. In contrast, in this present study, the completely random sequence RNAs were incubated in emulsion from the first selection round on, to identify RNAs generating a freely diffusing reaction product. The study thereby directly selected for a small metabolic system, in which two ribozymes—the GTP synthase and the polymerase ribozyme—were metabolically coupled through their common metabolite GTP.

The selected ribozyme GTR1 catalyzes the triphosphorylation of free guanosine and generates free GTP. This is a challenging task for a ribozyme because the ribozyme needs to bind two small-molecule substrates, catalyze their reaction, and release the product. The catalytic rate enhancement of 18,000-fold suggests that the first three of these requirements are met well. The low turnover number of 1.7-fold may originate from a slow product release. A slow product release could be caused by the requirement during the selection to bind guanosine and cTmp such that the produced GTP may have become bound tightly at the catalytic site.

The choice of guanosine—or specifically, 6sGsn—as substrate for the in emulsio selection was based on (i) the successful previous use of 6sGsn in an in vitro selection based on the gel shift in an APM polyacrylamide gel (*31*), (ii) the commercial availability of the thio-modified nucleoside, and (iii) the fact that guanosine provides better stacking than the otherwise also useful 4-thio uridine (*45*). Nature’s choice for guanosine as external substrate in group I intron ribozymes showed that guanosine is well suited to be bound tightly and specifically and then serve as reactant (*46*). However, guanosine is harder to activate at its 5′-hydroxyl group (*21*) and its 2′- and 3′-hydroxyl groups than the other canonical nucleosides (*47*). The slower uncatalyzed rate for guanosine to react with cTmp appears to stem, at least in part, from the tendency of the guanine base to occupy the anti-conformation in the nucleoside (*21*). On the basis of these low rates for the uncatalyzed reactions, GTP may have been in shorter supply than the other NTPs for an early life form. Therefore, a ribozyme generating GTP from guanosine and cTmp would likely have had a large evolutionary benefit. While our results showed that ribozyme-generated GTP can be incorporated into ribozyme-generated RNA polymers, the low turnover of GTR1 constrains the polymers that can be generated with the current ribozyme. We anticipate that future, improved versions of GTR1 will be able to supply GTP with an increased turnover and that additional nucleoside triphosphorylation ribozymes will also generate ATP, CTP, and uridine 5′-triphosphate (UTP) to allow ribozyme-mediated synthesis of RNA polymers from nucleosides and cTmp.

## MATERIALS AND METHODS

### Generation of the pool

A 188-mer DNA pool consisting of a 150-nucleotide randomized region flanked by PCR primer constant regions was ordered commercially [Integrated DNA Technologies (IDT)], with the sequence 5′-CAACATCACCGCCTTGTA-N150-GGGATTCTCCAGAACCTCGC-3′. Because only 2.2% of the pool was amplifiable by quantitative PCR, we assumed an upper limit on the pool complexity at 1.6 × 10^14^. PCR amplification (45 ml) used the constant regions as primer binding sites. Products were purified on commercial PCR clean-up columns (Macherey Nagel) and used as 30 nM template in a second PCR. During this PCR, primers added the T7 transcription promoter sequence and a hammerhead ribozyme sequence to the 5′ end of the DNA pool construct, and a DNAzyme recognition site to the 3′ end of the DNA pool construct. The hammerhead ribozyme would cleave cotranscriptionally to generate a free 5′-hydroxyl; this was done to prevent the possibility that pool molecules would use their own 5′-triphosphate to activate guanosine. The DNAzyme recognition site allows recognition and cleavage by a DNAzyme, which generates a free 3′-hydroxyl group. This 3′-hydroxyl can then be recognized by the polymerase ribozyme. This resulted in a final sequence of the DNA library of 5′-AATTTAATACGACTCACTATAGGGATGTTGCTGACGAGCTAAGCGAAACTGCGGAAACGCAGTCCAACATCACCGCCTTGTA-N150-GGGATTCTCCAGAACCTCGCTCGCGCATGTAAGTCTCACCAACTTATATGTTCTAGCGCGGA-3′, with underlined sequences acting as recognition sites for the DNAzyme (below). The PCR products were transcribed with T7 RNA polymerase and purified by denaturing 5% PAGE. A small amount of RNA pool was 5′ end radiolabeled using polynucleotide kinase (PNK) (New England Biolabs) and γ-[^32^P]–ATP and purified by denaturing 5% PAGE. The radiolabeled and unlabeled RNA pool were combined and processed together during the subsequent DNAzyme reaction.

### DNAzyme reaction

The RNA pool molecules were processed at their 3′-termini with a DNAzyme to generate a defined 3′-end with 2′,3′-hydroxyl groups. While 2′,3′-cyclic phosphates could have been generated much easier by self-cleaving ribozymes at the 3′-terminus, this was not done because of a possible selection artifact: Because 2′,3′-cyclic phosphates are chemically activated phosphates that can be used to drive the formation of a phosphodiester bond (see the reversibility of hairpin ribozyme reactions), they could also have led to the selection of a ligase ribozyme that catalyzes the nucleophilic attack of the 5′-hydroxyl group of 6sGsn to the 2′,3′-cyclic phosphate and thereby tag the RNA pool molecule with 6-thio guanosine 5′-monophosphate (6sGMP). While it might have been possible to remove 2′,3′-cyclic phosphates with alkaline phosphatase, we were concerned that some pool molecules might have been able to hide their 3′-termini from alkaline phosphatase. Therefore, the most reliable solution appeared to be processing by the DNAzyme. The DNAzyme reaction was a modified protocol for DNAzyme 9SK17, which was previously selected to cleave RNAs by generating a 5′-phosphate and 2′,3′- hydroxyl groups (*26*). The DNAzyme sequence was 5′-GCGCTAGAACATGCCAGCGATCAAAGACGGCGAGTTGTACCCATAGGTGTCTAGTTGGTGAGACTT-3′, where the underlined sequences were complementary to the 3′-constant region of the RNA library. A competitor DNA (5′-GCGAGGTTCTGGAGAATCCC-3′), complementary to the pool molecule upstream of the DNAzyme binding site, was used to mitigate inhibition of cleavage. The nucleic acids were prepared by heat renaturing 4 μM pool RNA, 8 μM DNAzyme, and 6 μM competitor DNA in 5 mM Hepes-NaOH (pH 7.5), 15 mM NaCl, and 0.1 mM EDTA and immediately placing them on ice for 10 min. An equal volume of reaction buffer was added to give final concentrations of 70 mM Hepes-NaOH (pH 7.5), 150 mM NaCl, 1.25 mM ZnCl_2_, 15 mM MnCl_2_, and 10 mM MgCl_2_. The ZnCl_2_ for the reaction buffer was prepared as an 8× stock from ZnCl_2_ powder, by adding in order the compounds to give final concentrations of 10 mM ZnCl_2_, 20 mM HNO_3_, and 200 mM Hepes-NaOH (pH 7.5). The reaction was incubated at 37°C for 3 hours. The reaction was stopped by adding a stoichiometric excess of EDTA and a DNA oligonucleotide that is reverse complementary to the competitor DNA (5′-GGGATTCTCCAGAACCTCGC-3′) in formamide loading buffer and purified using denaturing 5% PAGE.

### Selection step in emulsion

The oil phase consisted of 4% ABIL EM 90 (v/v) with 96% (v/v) heavy mineral oil. The oil phase was stirred at room temperature and then degassed in oil vacuum until no bubbles were present and stirred until the aqueous phase was prepared. To prepare the aqueous phase (2.5 ml in a 50-ml emulsion), RNAs were incubated with 100 mM tris-HCl (pH 8.3) and heat-denatured using a thermocycler at 80°C for 2 min before being immediately chilled on ice for 5 min. The RNAs were then mixed with an equal volume of buffer containing MgCl_2_, KCl, cTmp, and 6sGsn, resulting in final concentrations of 3 μM polymerase ribozyme, 0.5 μM RNA pool, 50 mM tris-HCl (pH 8.3), 150 mM MgCl_2_, 200 mM KCl, 50 mM cTmp, and 1 mM 6sGsn (later selection rounds contained less cTmp and 6sGsn). The reaction was started by quickly mixing the aqueous phase while on ice and then adding the entire mixture to the stirring oil phase. This generated a raw emulsion, which was mixed for 30 s before loading into the microfluidizer.

The emulsion was passed through a microfluidizer (Microfluidics, M110L) seven times at a pressure of 6000 psi (flow cell H10Z). The emulsion was then incubated at room temperature for 6 hours. The droplet size of the emulsion was measured by dynamic light scattering (DLS). In summary, a neodymium laser beam (532 nm) was passed through a 300-fold dilution of the emulsion in water-saturated oil phase. Light scattered at 90° was detected by a photomultiplier tube specially designed to tolerate large photon count rates. The measurements used a 0.5-ms dwell time in a multichannel scalar (FAST ComTec GmbH). Autocorrelation functions were calculated offline and fit to obtain droplet diameters as illustrated in Fig. 2B for one typical case.

The incubated emulsion was processed by first adding a molar excess of EDTA to chelate Mg^2+^ ions, followed by addition of 5 ml of water to increase the volume of the aqueous layer. The addition of 5 ml of diethyl ether then broke the emulsion. To isolate the RNA, the emulsion was centrifuged at 15,000*g* for 30 min at 4°C. The aqueous phase was extracted with an excess of diethyl ether and centrifuged again. The pellet was vortexed with 5 ml of phenol until it dissolved, and centrifuged at 15,000*g* for 10 min at 4°C. The aqueous phase was extracted with an excess of chloroform and centrifuged at 15,000*g* for 5 min at 4°C. RNA was recovered from the aqueous layer by ethanol precipitation.

To purify active sequences by 5% APM-PAGE, the polyacrylamide gel was made in three layers. The bottom and top layers both consisted of 5% polyacrylamide. The middle layer contained 0.1 mM APM (from a stock solution of 3 mM APM in *N*,*N*′-dimethylformamide). The sample was loaded in the middle of the gel alongside a positive control that consisted of RNA pool spiked with 6sGTP. After electrophoresis for 4 to 6 hours, RNA sequences at the APM interface were visualized using the 5′ radiolabeled pool molecules introduced during the DNAzyme processing step. The interface band was excised and eluted in 300 mM NaCl and 5 mM DTT overnight at 4°C. Following ethanol precipitation, the pool molecules were reverse-transcribed using SuperScript III and the RT primer 5′-GCGAGGTTCTGGAGAAT-3′. Reverse transcriptase (RT) products were PCR-amplified using the same primers given above to generate the selection library, completing one round of selection.

### HTS analysis

PCR products were barcoded for each selection round and sequenced on an Illumina MiSeq machine using paired-end 250 sequencing (UCSD IGM Genomics Center). The output of MiSeq data was given as a demultiplexed FASTQ file format. The data were uploaded to the online Galaxy bioinformatics website, where the constant regions were clipped and the files were converted to FASTA format. Each round of selection contained its own FASTA file sorted by sequence abundance. Sequence clusters were generated in the USEARCH suite (*48*), with an identity threshold of 0.75 in the fast function. Clustering was done using a greedy algorithm, and so, the cluster centers became defined as the most abundant sequence due to the sequence order within the FASTA file. A combination of Python, terminal commands, and Excel was used to track the abundance of each cluster through the rounds of selection. An R script was used on each cluster to track the progression of individual sequences within each cluster through each round. Abundant sequences were aligned using the MUSCLE suite (*49*). This allowed the identification for highly enriching mutations within a cluster.

### Guanosine triphosphorylation assay

The guanosine triphosphorylation assay follows the same principle as the reaction in emulsion during the selection procedure, but using the final concentrations 1 μM GTR, 0.5 μM radiolabeled primer (5′-CUCACCAACU-3′), 1 μM polymerase ribozyme, 0.5 mM 6sGsn or 1.0 mM Gsn, 25 mM cTmp, 50 mM tris-HCl (pH 8.3), 150 mM MgCl_2_, and 200 mM KCl. To initiate the assay, the GTR was heat-renatured in 50 mM tris-HCl (pH 8.3) by incubating at 80°C for 2 min and then allowed to cool at room temperature for 5 min. A premix containing the polymerase ribozyme and radiolabeled primer was heat-renatured in 50 mM tris-HCl (pH 8.3) according to the same heating profile. The guanosine triphosphorylation ribozyme premix and the polymerase ribozyme premix were added together in equal volume. A reaction premix containing tris-HCl, MgCl_2_, KCl, cTmp, and 6sGsn was then added to the ribozyme mix in equal volume. The reaction was incubated at room temperature for 6 hours. Products were mixed with a loading buffer containing 40% (v/v) formamide and 1.5 μM final concentration of a DNA oligomer complementary to the RNA primer. After heat renaturing (2 min at 80°C), the samples were separated by 7 M urea 20% PAGE. The separations were exposed to phosphorimager screens and scanned with a phosphorimager (Typhoon NIR Plus, Amersham), and bands were quantified using Quantity One software. The data were processed in Excel worksheets.

For the optimization of reaction conditions, the guanosine triphosphorylation assay was modified to quench GTR1 catalysis at specific time points and thereby allow measuring reaction kinetics. All reactions were performed at room temperature. A premix was made as follows: 1 μM ribozyme in 50 mM tris-HCl (pH 8.3) was incubated with MgCl_2_, KCl, cTmp, and Gsn at the indicated concentration. For the titrations of MgCl_2_, KCl, cTmp, Gsn, and ribozyme, only one component was variable, while the rest were held constant. For the pH optimization, different buffers were used: tris-HCl (pH 9.0, 8.5, and 8.0), Hepes-NaOH (pH 7.0), and MES/NaOH (pH 6.5 and 6.0). At a given time point, the reaction was quenched by adding a mix of three DNA inhibitors (fig. S5), each at a 19.4-fold molar excess over GTR1 (GTR1 at 1 μM in the reaction). The excess was lower at higher GTR1 concentrations (twofold at 9 μM GTR1).

To determine reaction kinetics under optimized conditions, GTP generated by the GTR was used by a polymerase ribozyme to extend a substrate RNA by one nucleotide. A mixture containing RNA primer (5′-CUCACCAACU-3′, 0.5 μM final), trace-radiolabeled RNA primer, and polymerase ribozyme (1 μM final) was added to the GTR mix. The mixture also included KCl such that the overall KCl concentration was maintained at 200 mM, and it included tris-HCl (pH 8.3) such that the concentration of tris-HCl was maintained at 50 mM. In addition, the magnesium concentration was 150 mM in the polymerase ribozyme reaction regardless of magnesium concentration in the GTR mix. If the concentration of magnesium after mixing the GTR mix and the polymerase ribozyme mix was greater than 150 mM, excess magnesium was chelated using EDTA to bring the concentration of free Mg^2+^ down to 150 mM. If the concentration of magnesium after mixing the GTR mix and the polymerase ribozyme mix was less than 150 mM, additional magnesium was added to bring the concentration up to 150 mM. Reaction products were mixed with a loading buffer containing 40% (v/v) formamide and 1.5 μM final concentration of a DNA oligomer complementary to the RNA primer. After heat renaturing (2 min at 80°C), the samples were separated by 7 M urea 20% PAGE.

The separations were exposed to phosphorimager screens and scanned with a phosphorimager (Typhoon NIR Plus, Amersham), and bands were quantified using Quantity One software. The data were processed in Excel worksheets. Single exponential fits were adjusted to all kinetic data (each with five time points 5, 20, 80, 320, and 1280 min) using the Excel subroutine Solver to the equation “fraction ligated” = A*(1 − EXP(−*k*_OBS_*t)) + *C*, where fraction ligated is the experimentally determined fraction of 10-mer RNA primer extended by the polymerase ribozyme, *t* is triphosphorylation time, *A* is the amplitude of the curve, *k*_OBS_ is the observed rate, and *C* describes the constant background.

### Truncation of the winning isolate

The sequence C0-59 was chosen as the best initial isolate from the in vitro selection. A series of 5′, 3′, and internal truncations were made and tested for activity. 5′ and 3′ truncations were generated by PCR using Taq DNA polymerase and a series of different 5′ and 3′ primers for each truncation. DNA templates of each truncation were transcribed using T7 RNA polymerase and gel-purified. The truncated ribozymes were then tested for activity using the previously described guanosine triphosphorylation assay. None of the 5′ or 3′ truncations were active.

A series of truncations, as well as two combinations of internal truncations, was made (fig. S4). DNA templates containing the internal truncations were obtained commercially (IDT) as gBlocks. Plasmids for each truncation were generated by cloning into pUC19 and confirmation by sequencing. DNA templates of each truncation were generated by PCR from each plasmid, transcribed using T7 RNA polymerase, and PAGE-purified. The truncated ribozymes were then tested for activity using the guanosine triphosphorylation assay described above.

### Secondary structure probing using SHAPE

SHAPE was performed on GTR1 using previously published methods (*37*). A 20-nucleotide extension that did not interfere with ribozyme function was added to the 3′ end of the ribozyme by PCR amplification. The sequence of this primer is (5′-ACATATCGTCGGAGCCATTGGAGACTTACATGCGCGAG-3′), where the underlined portion is complementary to the ribozyme. 1M7 was used as the chemical probe. Ribozyme (90 pmol) was heat-renatured at 80°C for 2 min, cooled to 50°C for 5 min, and then left at room temperature for 5 min. To this solution, a final concentration of 50 mM Hepes-NaOH (pH 8.0), 2 mM Gsn, 1 M KCl, and 300 mM MgCl_2_ was added. This mixture was incubated at room temperature for 2 min. A 100 mM stock solution of 1M7 was prepared in dimethyl sulfoxide (DMSO) and added as ^1^/_50_ of the ribozyme solution, resulting in final concentrations of 2 mM 1M7 and 2% (v/v) DMSO. A control without 1M7 was prepared with ribozyme, Hepes, Gsn, KCl, MgCl_2_, and 2% (v/v) DMSO. Both samples were incubated at room temperature for 3 min. The reaction was quenched by ethanol precipitation and resuspended in 10 μl of 5 mM tris-HCl (pH 8.0). The products were reverse-transcribed using SuperScript III reverse transcriptase (Invitrogen) according to the manufacturer’s instructions and trace amounts of a radiolabeled primer that annealed to the aforementioned 3′-extension of the ribozyme. The sequence of this primer is (5′-ACATATCGTCGGAGCCATTG-3′). It was difficult to resolve products larger than 60 nucleotides in length using this primer, so the protocol above was repeated using one additional radiolabeled primer during the reverse transcription step. The other primer (5′-GTATGATACAGGCGAGGC-3′) annealed to positions 65 to 82 of the ribozyme. Following the reverse transcription, the RNA template in each sample was degraded by alkaline hydrolysis. To do that, the samples were incubated for 5 min at 80°C in a solution containing 750 mM NaOH. The reaction was quenched by addition of acetic acid to a final concentration of 300 mM NaOAc/HOAc (pH 5). Then, the products were ethanol-precipitated, resuspended in formamide loading buffer, and resolved by 7 M urea 20% PAGE.

### MS identification of GTP

A reaction mixture consisting of 50 mM tris-HCl (pH 8.0), 200 mM KCl, 125 mM MgCl_2_, 1 mM Gsn, 25 mM cTmp, and 9 μM GTR1 was incubated at room temperature for 24 hours. GTR1 was heat-renatured in water for 10 min at 50°C and then cooled for 5 min at 22°C before the other compounds were added. A mixture containing water in place of GTR1 was used as a negative control, and a sample containing 6 μM GTP in place of GTR1 was used as a positive control. Twenty-five microliters of each sample was analyzed by LC-MS. Twenty microliters of 6 μM GTP in water was used as standard. After incubation, each sample was centrifuged at 10,000*g* for 5 min to remove possible particles, and the clear supernatant was submitted for LC-MS [University of California San Diego (UCSD) facility].

Samples were loaded on a 150-mm-long C18 reversed-phase column at 37°C (Scherzo SM-C18, metal-free column, Imtakt USA) in 50 mM ammonium formate (pH 8.6) and eluted in a gradient over 10 min (3 ml) with 100 mM ammonium formate (pH 8.6). Elution profiles were recorded at 260 nm with 4-nm bandwidth. The HPLC system (Agilent 1260 Infinity) was coupled to a Thermo LCQdeca MS, which used positive ion mode electrospray ionization as the ion source. The source voltage was 5 kV, the sheath gas rate was 80 units, the auxiliary gas flow was 20 units, and the capillary gas temperature was 250°C.

### Ribozyme-mediated RNA polymerization

Ribozyme-mediated RNA polymerization was performed essentially as described (*7*), but the GTP was supplied from a preceding reaction between guanosine and cTmp. Specifically, 9 μM of ribozyme GTR1 was incubated with 50 mM tris-HCl (pH 8.0), 200 mM KCl, 125 mM MgCl_2_, 25 mM cTmp, and 1 mM Gsn at 22°C for 24 hours. These conditions are not the optimal conditions for GTR1 but were adjusted to work together with the polymerase ribozyme. The polymerase ribozyme 8.12.23 was heat-renatured 2 min at 80°C in water together with RNA template 5′-GACGCUUCGCACGGUUGGCAG-3′, 5′-[^32^P]–radiolabeled RNA primer 5′-CUGCCAACCGUG-3′, and P2 oligonucleotide 5′-GGCACC-3′. The reaction was started by combining the GTR1 reaction mixture, RNAs, and a premixed buffer to result in the final concentrations of 2.5 μM P2 oligonucleotide, 2 μM polymerase ribozyme, 1 μM template, 0.5 μM primer, 10 μM CTP, 30 μM UTP, 30 μM ATP, 50 mM tris-HCl (pH 8.3), 200 mM MgCl_2_, and 50 mM KCl, with one-quarter of the reaction volume being the GTR1 reaction mixture. The reaction was incubated at 20°C for the given times and quenched by adding a final concentration of 40% (v/v) formamide, a molar excess of Na_2_EDTA over the Mg^2+^ in the reaction, a 20-fold excess of an RNA complementary to the template, and heat denaturation 2 min at 80°C. The separations were exposed to phosphorimager screens and scanned with a phosphorimager (Typhoon NIR Plus, Amersham), and bands were quantified using Quantity One software. The data were processed in Excel worksheets.
