# Supplementary material for: A GTP-synthesizing ribozyme selected by metabolic coupling to an RNA polymerase ribozyme
Source: Sci Adv. 2021 Oct 6;7(41):eabj7487. doi: 10.1126/sciadv.abj7487 (PMC8494290; doi:10.1126/sciadv.abj7487)
Supplement: Supplementary file 1 — Figs. S1 to S7 [file sciadv.abj7487_sm.pdf]

Science Advances
AAAS

Supplementary Materials for

A GTP-synthesizing ribozyme selected by metabolic coupling to an RNA polymerase ribozyme

Arvin Akoopie, Joshua T. Arriola, Douglas Magde, Ulrich F. Müller*

*Corresponding author. Email: ufmuller@ucsd.edu

Published 6 October 2021, *Sci. Adv.* **7**, eabj7487 (2021)
DOI: 10.1126/sciadv.abj7487

This PDF file includes:

Figs. S1 to S7

Figure S1

A

Round	[cTmp] mM	[6sGsn] mM	[RNA pool] nM
1	50	1	500
2	50	1	500
3	25	0.5	500
4	12.5	0.25	500
5	12.5	0.25	500
6	12.5	0.25	500
7	12.5	0.25	500
8	12.5	0.25	500
9	12.5	0.25	500
10	12.5	0.25	100
11	12.5	0.25	100
12	12.5	0.25	100
13	2.5	0.1	100
14	1	0.05	50
15	2.5	0.1	25
16	2.5	0.1	10
17	0.5	0.05	10
18	0.5	0.05	10

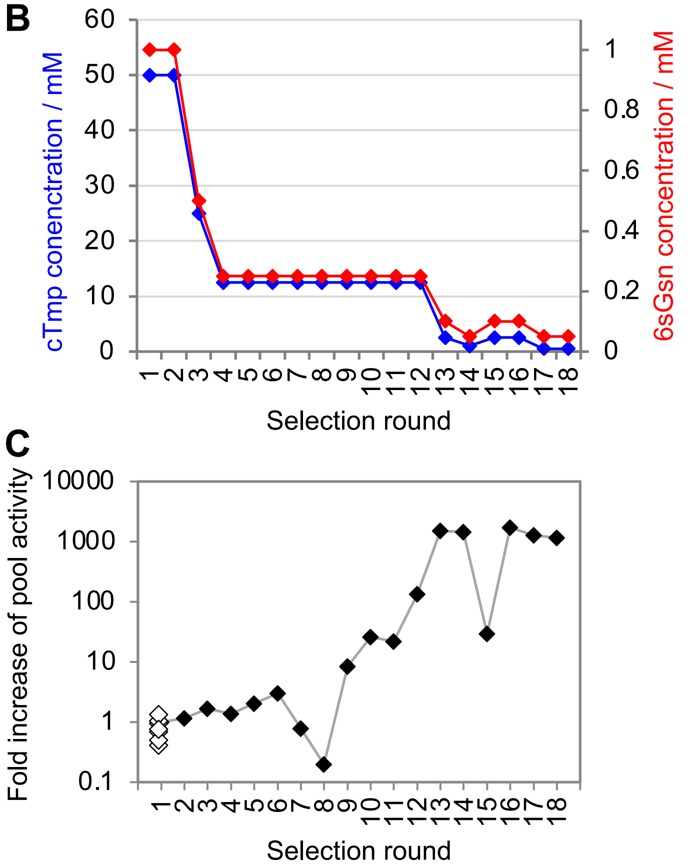

Figure S1. Parameters that were adjusted over the selection rounds. **(A)** For each selection round (left column), the concentrations of cTmp and 6sGsn in the aqueous phase of the emulsion are given in mM, and the concentration of RNA library in nM. Note that the concentration of the polymerase ribozyme stayed constant at 3 micromolar. While only a stoichiometry of 1:1 would be needed to anneal to all pool molecules in bulk, the total concentration of 3 micromolar made sure that about 95% of the emulsion droplets contained at least one polymerase ribozyme molecule, to allow tagging of the pool molecules with produced 6sGTP. **(B)** Plot of the two adjustments affecting the selection pressure, cTmp concentration and 6sGsn concentration, over the selection rounds. **(C)** Plot of the normalized pool activity over the selection rounds. In contrast to figure 2C, the pool activity was plotted logarithmically. The drop in activity in selection rounds 7 and 8 was caused by a selection artifact that appeared to form dimers of the RNA pools. These artifacts were successfully removed by increasing the time for electrophoresis during APM-PAGE purification of active pool molecules. The logarithmic plot emphasizes minor changes in pool activity over earlier selection rounds, and thereby makes clear why parameters in the selection conditions were changed at given rounds.

Figure S2

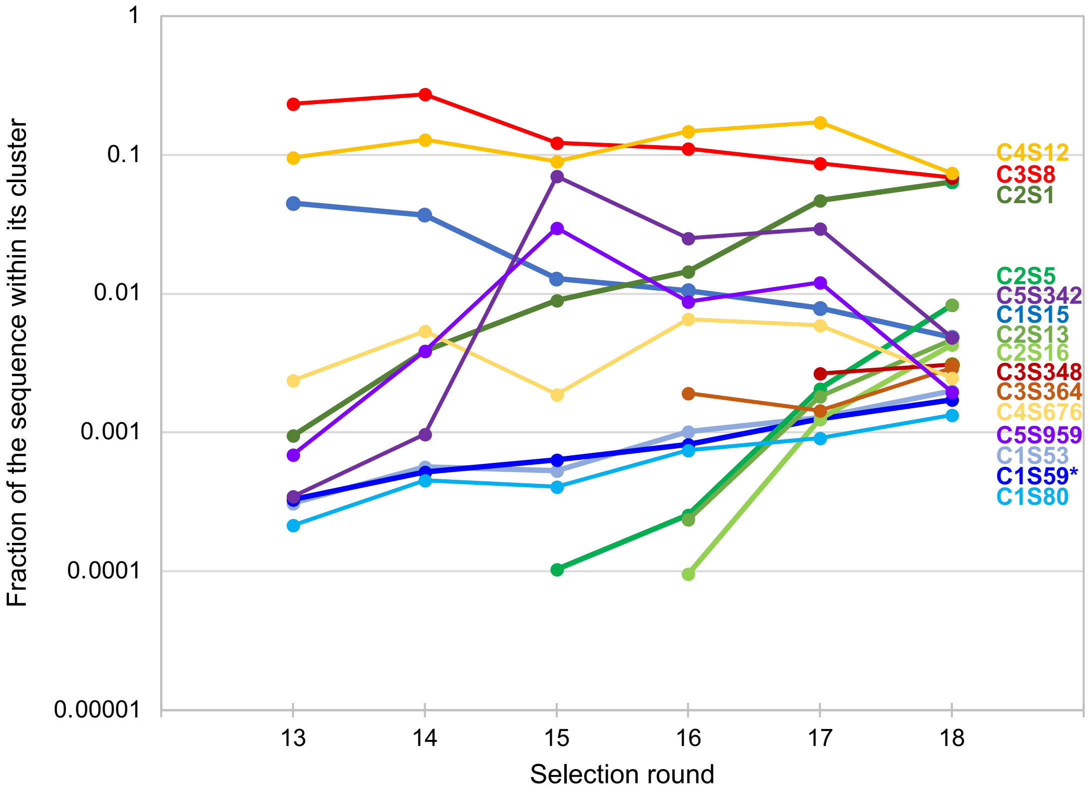

Figure S2. Abundance of individual sequences in the High Throughput Sequencing data for rounds 13 to 18 of the selection. The abundance of each sequence *within its cluster* is plotted as a function of the selection round. The name of the sequence is given on the right, in the same color as the corresponding data set. The colors correspond to the colors used for each cluster in figure 2B, with cluster 1 blue, cluster 2 green, cluster 3 red, cluster 4 yellow, and cluster 5 purple. Different shades of these colors are used to discriminate individual sequences with a given cluster. The sequence with highest biochemical activity for GTP synthesis (cluster 1 sequence 59) is marked with an asterisk, and steadily increased 5.2-fold in frequency from round 13 to 18. The two sequences that showed highest biochemical activity for 6sGTP synthesis (C5S342 and C5S959; see figure S3) also showed the highest enrichment factor in the earlier rounds R13 to R15 (200-fold and 43-fold, respectively) but decreased in abundance after mutagenic PCR was used in round 15.

Figure S3

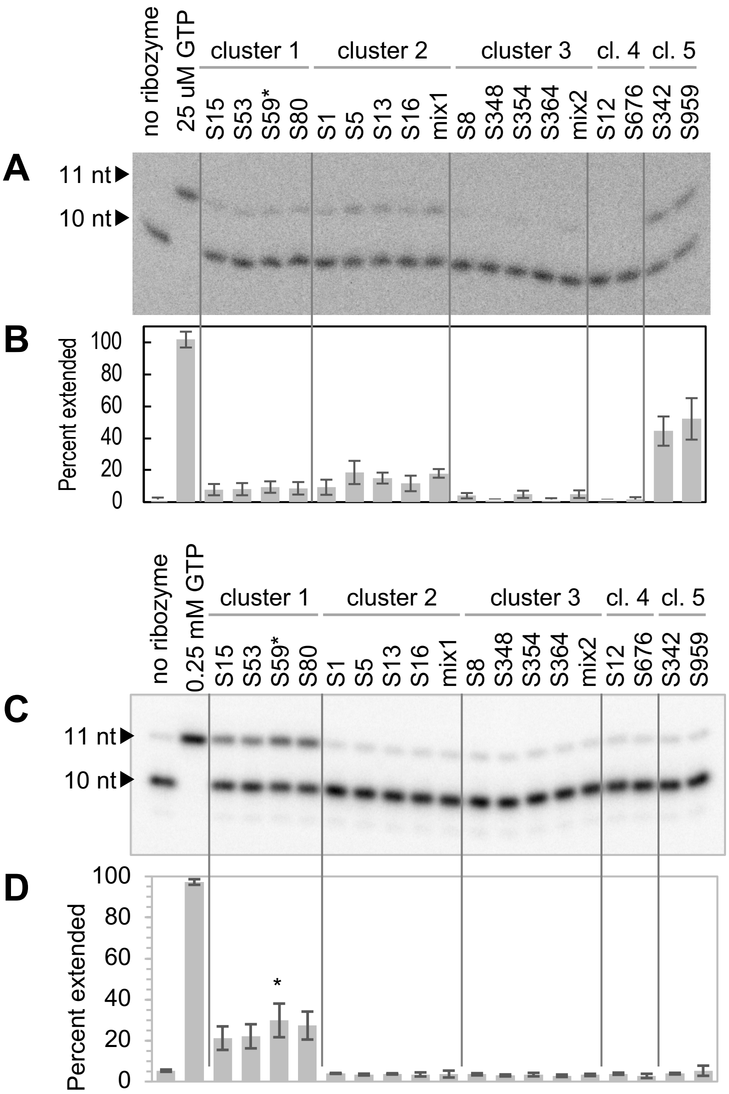

Figure S3. Biochemical assay for nucleoside triphosphorylation activity. The assay was conducted as shown in figure 3A. **(A)** Autoradiogram of reaction products after separation by denaturing 20% PAGE. Individual sequences (S1-S959) from five sequence clusters were tested alongside a positive control (0.25 mM GTP) and a negative control (no ribozyme). A 10 nucleotide long 5'-radiolabeled radiolabeled primer was extended with the produced 6sGTP, utilizing a polymerase ribozyme. The resulting gel shift can be seen in the autoradiogram. **(B)** Analysis of the signals in (A). The percent of radioactive primer that was extended from 10 nt to 11 nt is shown as the average of three replicates. Error bars are standard deviations from three replicates. Ribozyme clones from clyster 5 show the highest activity, followed by ribozymes from cluster 2 and cluster 1. **(C)** The same analysis as in (A) but with guanosine instead of 6-thio guanosine, monitoring the production of GTP instead of 6sGTP. **(D)** Analysis of the signals in (C), as in (B). Ribozyme clones from cluster 1 showed the highest activity. The ribozyme clone S59 in cluster 1 showed the highst average activity and was chosen for further analysis.

The assay was so sensitive that the background reaction between 6sGsn and cTmp at near-neutral pH led to detectable signal. This sensitivity emerged because (i) the assay employed on the order of 1,000-fold more 6sGsn (mM) than pool molecules (mM), because (ii) even micromolar concentrations of 6sGTP are sufficient to efficiently extend the radiolabeled RNA 10-mers (*27*), and because (iii) the shift of 0.1% of the bands in PAGE analysis was within the quantifiable range.

Figure S4

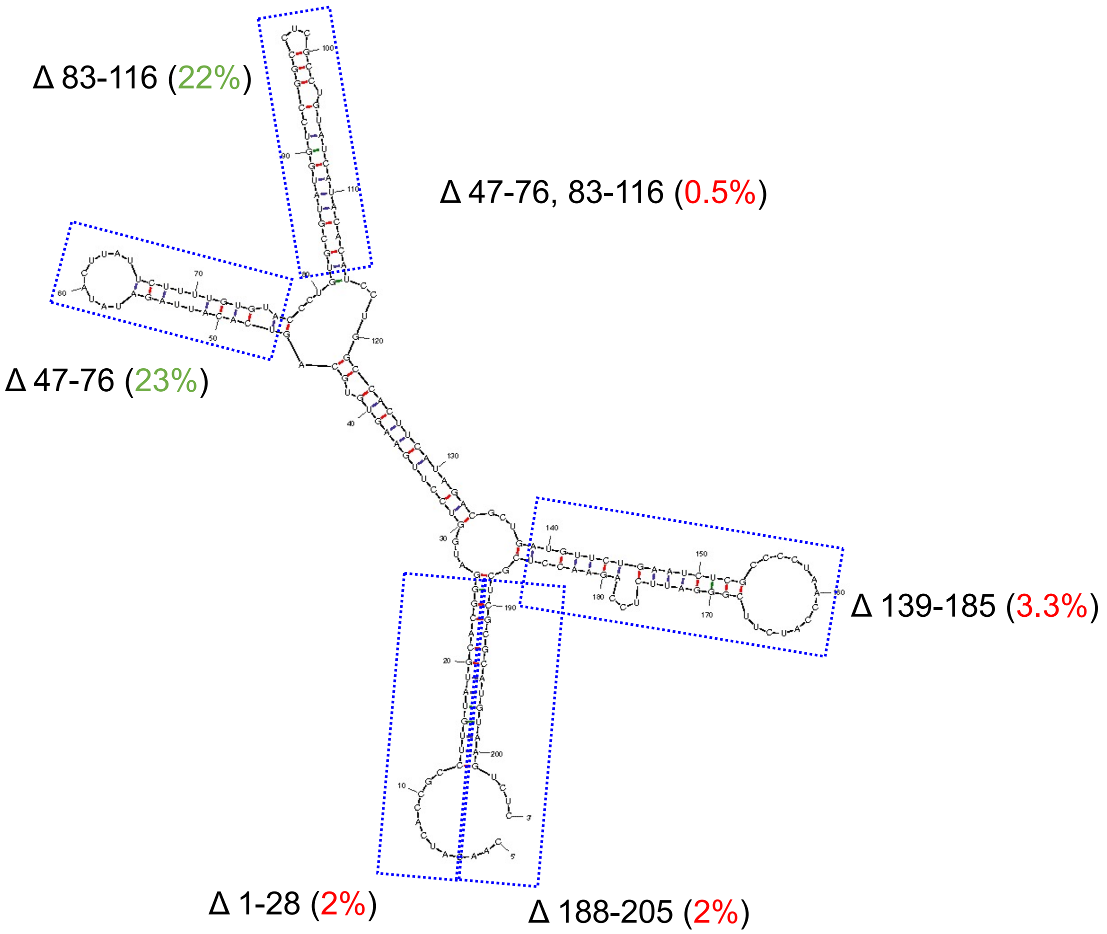

Figure S4. Truncation analysis of the most active isolate from the selection, cluster 1 clone 59. The activities of different truncation constructs are projected on a preliminary secondary structure (Zuker 2003, Nucl. Acids Res 31, 3406). The deleted regions in the ribozyme are shown in blue boxes for each deletion construct. The activities using the trans-assay (figure 3) are included with the label of each deletion variant. The full-length ribozyme generated enough GTP so that 25% of the radiolabelled primer were extended. The values of 22% and 23% were within experimental error of the 25% activity measured for the wild-type. The results suggested that the regions 47-76 and 83-116 were individually dispensable but not in combination. All other tested regions appeared essential to the ribozyme.

Figure S5

A

GTR1:
CAACATCACCGCCTTGTATGCACGGGATGGTCCTTGAAGTGTGCAGCCCTGTGCGTATGGTCC
GGCCTCGCCTGTATCATACACATCCTGGCCACTTCATAGACGCTGATGTTCTGAATCTCGCCC
CTAACCATCTTCGGGATTCTCCAGAACCTCGCTCGCGCATGTAAGTCTC-3'

D60.198 (anneals to position 1 – 60):
CCATACGCACAGGGCTGCACACTTCAAGGACCATCCCGTGCATACAAGGCGGTGATGTTG-3'

D60.199 (anneals to position 70 – 130):
TAGGGGCGAGATTCAGAACATCAGCGTCTATGAAGTGGCCAGGATGTGTATGATACAGGC-3'

D46.32 (anneals to position 131 – 175):
GAGACTTACATGCGCGAGCGAGGTTCTGGAGAATCCCGAAGATGGT-3'

B

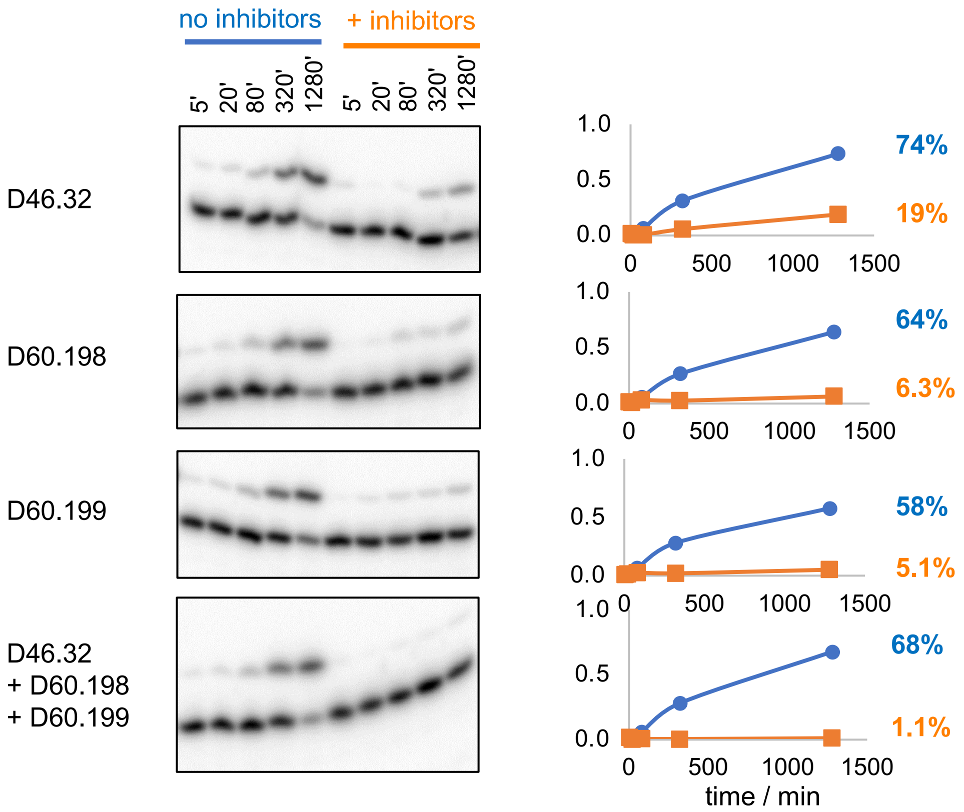

Figure S5. Quenching the activity of the guanosine triphosphorylation ribozyme GTR1 using complementary DNA oligonucleotides. **(A)** sequence of the GTR1 ribozyme and each of the three inhibitor oligonucleotides, with the complementarity indicated in color. **(B)** Activity of the ribozyme with and without each of the inhibitors. For each inhibitor, or combination of inhibitors, a triphosphorylation time course was measured with over 21 hours (1280 min). Because each individual inhibitor allows >5% of activity within 21 hours, the combination of all three DNA oligonucleotides D60.198, D60.199, and D46.32 was chosen to quench the activity of GTR1 in the assays for the optimization of reaction conditions, and the measurement of the catalyzed rate.

Figure S6

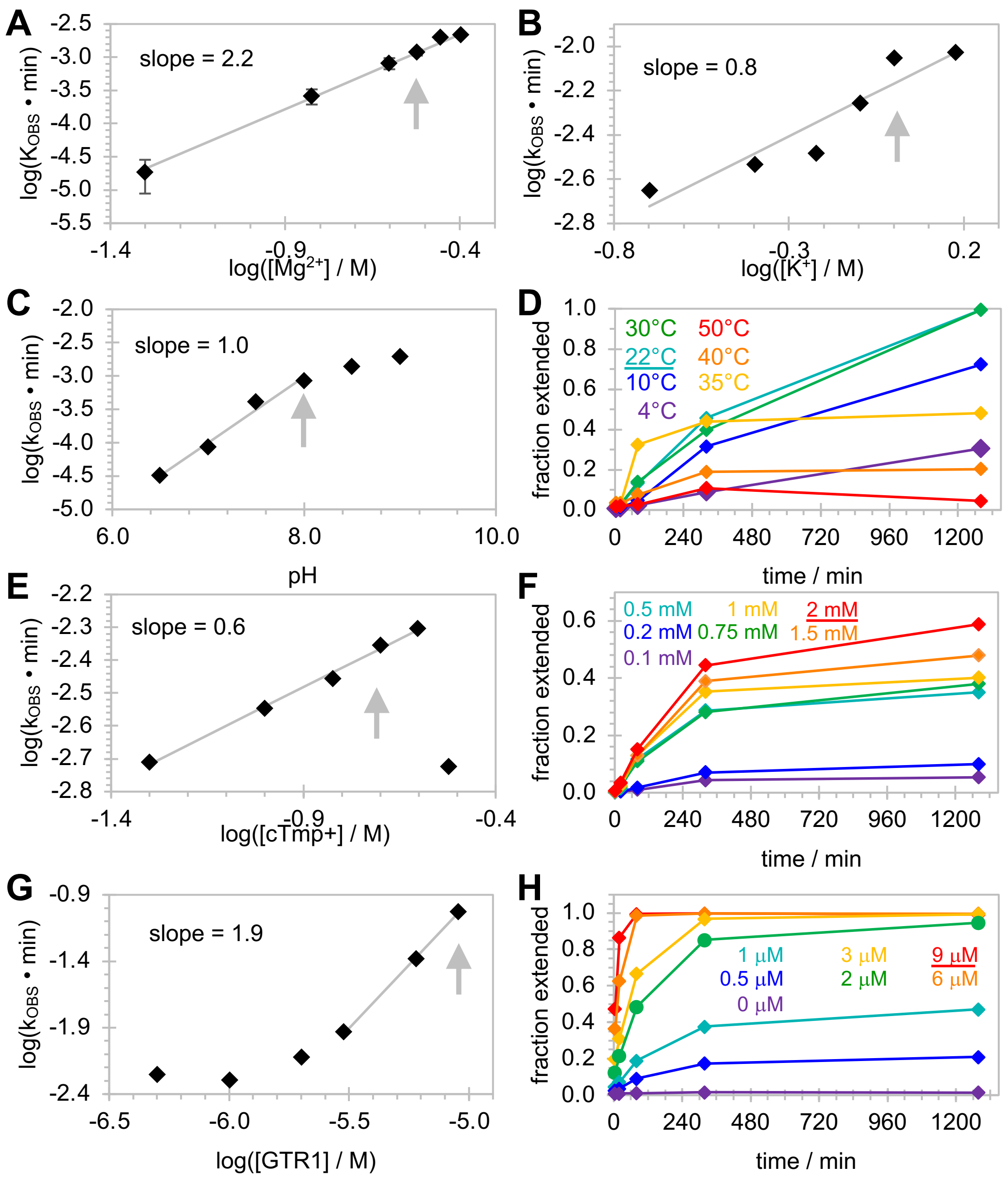

Fig. S6. Optimization of reaction conditions for GTR1. The reaction rates were determined by single-exponential curve fitting to reaction time courses and plotted as function of the reaction conditions. The condition in one assay was then used for the next assay. Starting from an initial condition of 125 mM Mg^{2+}, 100 mM KCl, 50 mM Tris/HCl pH 8.3, 22°C, 25 mM cTmp, 1 mM Gsn, and 1 μM ribozyme the conditions were successively optimized to **(A)** 300 mM Mg^{2+} (error bars are standard deviations from three experiments), **(B)** 1 M K^+, with a 95% confidence interval of +/-0.3 for the slope of 0.8, **(C)** pH 8.0, **(D)** 22°C, **(E)** 200 mM cTmp, **(F)** 2 mM guanosine, and **(G,H)** 9 μM GTR1. The sigmoid dependence on the GTR1 concentration suggested that at low GTR1 concentration a portion of the ribozyme was inactive. On the other hand, at GTR1 concentrations above 2 μM ribozyme catalysis slowed but a larger fraction of the ribozyme folded into the active conformation. Because high concentrations of KCl, $MgCl_2$, and cTmp led to precipitation the chosen 'optimal conditions' do not always represent the highest points in the graphs. Panels D, F, and H are shown as kinetic plots to illustrate the reduced amplitude at certain conditions. Gray arrows and underlined legends indicate the chosen conditions. The gray lines show linear least-squares fits to data points, with slopes given in the inserts. The data for the dependence on temperature, guanosine concentration and GTR1 concentration are shown as kinetic plots to clarify that the reaction amplitudes were sometimes significantly below 100% (panel D,F,H).

Figure S7

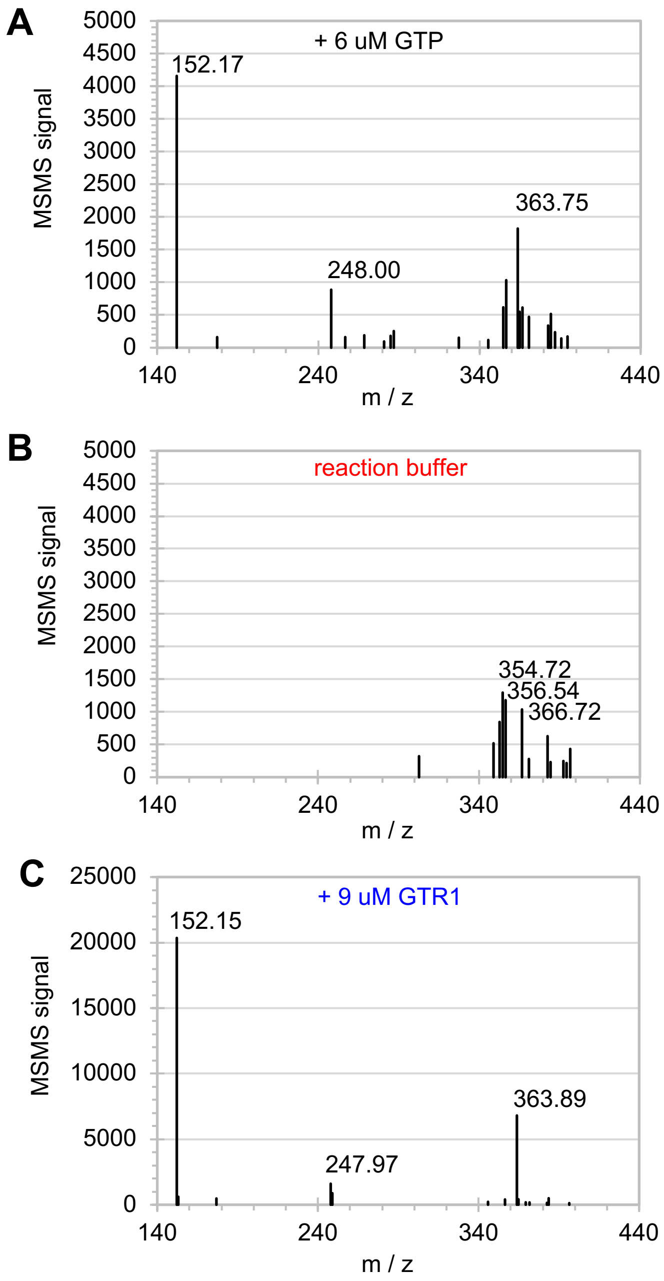

Figure S7: MSMS analysis of the fraction eluting at 1.9 minutes, the HPLC fraction where GTP showed a peak. **(A)** MSMS of reaction buffer containing 6 uM GTP. The characteristic signals are labeled. **(B)** MSMS of reaction buffer. Peaks in the vicinity of the characteristic GTP signals are labeled. No GTP characteristic signals were detected. **(C)** MS of the reaction with 9 uM GTR1. The three GTP characteristic signals are labeled.
